# Bridging the gap: optimizing conduction system pacing for cost-effectiveness and widespread adoption in low-resource settings

**DOI:** 10.2478/abm-2026-0018

**Published:** 2026-06-30

**Authors:** Ronpichai Chokesuwattanaskul, David Hayes

**Affiliations:** Division of Cardiovascular Medicine, Department of Medicine, Faculty of Medicine, Chulalongkorn University, King Chulalongkorn Memorial Hospital, Bangkok 10330, Thailand; Cardiac Center, King Chulalongkorn Memorial Hospital, Bangkok 10330, Thailand; Center of Excellence in Arrhythmia Research Chulalongkorn University, Department of Medicine, Faculty of Medicine, Chulalongkorn University, King Chulalongkorn Memorial Hospital, Bangkok 10330, Thailand; Department of Cardiovascular Diseases, Mayo Clinic, Rochester 55905, MN, USA

**Keywords:** cardiac electrophysiology, conduction system pacing, cardiac resynchronization therapy, pacing-induced cardiomyopathy

## Abstract

**Background:**

Right ventricular apical (RVA) pacing often leads to interventricular dyssynchrony and pacing-induced cardiomyopathy. Conduction system pacing (CSP), particularly Left Bundle Branch Area Pacing, provides a physiologic alternative. In Limited Resource Countries (LRCs) like Thailand, Cardiac Resynchronization Therapy (CRT) under-penetration persists due to high costs and a shortage of operators skilled in coronary venous anatomy.

**Objective:**

To share the experience and provide clinical update of CSP as a sustainable, cost-effective physiological pacing strategy to improve access to advanced arrhythmia management in resource-constrained environments.

**Methods:**

This review explores the implementation of minimalist protocols and CSP-based CRT. Scalability strategies include specialized training programs, device reprocessing, and the establishment of regional longitudinal registries to bridge infrastructure gaps.

**Results:**

CSP-based CRT can reduce device costs by nearly 50% compared with traditional triple-lead systems. This modality effectively mitigates the hemodynamic risks of RVA pacing while bypassing the technical and financial barriers of conventional CRT.

**Discussion:**

Despite its advantages, CSP adoption requires overcoming infrastructure challenges, specifically the lack of automated remote monitoring. Optimizing clinical outcomes in LRCs necessitates standardized training, robust data collection, and innovative resource management to ensure equitable access to life-saving technology.

## Physiological imperative of conduction system pacing

### Overview

Cardiac physiological pacing (CPP) is inclusive of biventricular pacing (BVP), Bachmann’s bundle pacing, and conduction system pacing (CSP). This review primarily focuses on left bundle branch area pacing (LBBAP) within the scope of CSP, as this method has the strongest evidence base to date. While pacing Bachmann’s bundle is a promising physiological technique for restoring atrial synchrony and enhancing atrial function, its clinical advantages must be more thoroughly validated before it can be recommended for broad implementation [1].

CSP maintains physiological activation of the heart and avoids traditional right ventricular apical (RVA) pacing-related complications. RVA pacing may contribute to interventricular dyssynchrony, meaning that the left ventricular septum and free wall contraction are discordant. This dyssynchrony increases the risk of heart failure (HF) from a resulting decline in left ventricular contractility. This adverse effect from RVA is termed “pacing induced cardiomyopathy” [2].

### Current international guidelines and indications for CSP

The existing evidence base for CSP is largely from observational studies. A limited number of small randomized controlled trials (RCTs) have been completed [3,4,5,6]. The observational data demonstrates a superiority over traditional conventional right ventricular (RV) pacing. In observational trials of CSP versus BiV pacing, the results have been mixed, showing non-inferiority of CSP when compared with BiV pacing, while in some studies superiority of CSP has been shown.

The recent Physio-Sync trial, performed in Brazil, was an RCT of CSP versus BiV. In this RCT, CSP was inferior to BiV pacing. Due to the lack of additional RCTs, many guidelines still preserve BiV pacing for indicated patients as the class I recommendation for physiological pacing, and CSP is recommended when cardiac resynchronization therapy (CRT) cannot be successfully accomplished in each patient [7].

Currently, the guidelines include CSP as part of the recommendations for CSP due to growing evidence of its benefits [7,8,9]. However, remaining concerns or unknowns include long-term lead durability and capture, ideal location for CSP lead position and extraction feasibility in long-term dwelling leads.

### CPP in atrioventricular block

Patients presenting with atrioventricular block (AVB) are the most clear-cut beneficiaries of CSP when compared with traditional RV pacing. The primary advantage of CSP is its ability to maintain near-physiologic ventricular activation, which minimizes the deleterious effects of non-physiologic RV pacing, notably the prevention of pacing-induced cardiomyopathy [10].

### CPP in CRT

In the setting of CRT-indicated patients, the clinical application of CSP requires rigorous patient selection. While promising, the current body of evidence includes a limited number of RCTs directly comparing CSP (e.g., LBBAP or his bundle pacing (HBP)) to traditional BVP. Patients most likely to achieve maximal CRT benefit from CSP often meet the following criteria:

- Typical left bundle branch block (LBBB) morphology.
- Absence of interventricular septal scar.
- No evidence of extensive myocardial disease.

A critical technical mandate for successful CRT via CSP is the strict confirmation of left bundle branch (LBB) capture to ensure effective ventricular resynchronization. This electrophysiological (EP) confirmation is paramount for clinical success.

## The burden of disease in limited resource countries

### Epidemiology of bradyarrhythmias and HF suitable for CSP/CRT in LRCs

The prevalence of conduction system diseases in Thailand is uncertain as we lack a good national health registry database. Based on the Thai EP club national registry from 42 centers nationwide, 4,000 pacemaker (1,300 CPP), 600 CRT, and 1,000 ICD are the estimated annual number of procedures that are growing in number [11]. This might imply from other registry data that in our indicated population for CPP, there is under-penetration of CRT. Notwithstanding the comparable prevalence of patients meeting the clinical criteria for CRT with heart failure with reduced ejection fraction population, current data suggests a significant therapeutic underutilization [12]. This deficit in CRT penetration is primarily attributed to clinical inertia regarding updated practice guidelines and systemic socioeconomic barriers, specifically restrictive reimbursement frameworks. Consequently, a substantial cohort of eligible candidates remain untreated despite established Class I indications [12].

### Reasons why standard BVP is often inaccessible or suboptimal in these regions

Given that BVP is the standard of care in HF patients with LBBB despite optimal medical therapy, it remains underused. Standard BVP requires special training skills to familiarize implanters with coronary venous anatomy. Operators across the region might not have sufficient time to invest in learning the necessary skills for this procedure. Low physician to population ratio and economic constraints are the main limitations to widely adopting BVP in this region. The cost is a main concern that limits the use of CRT as many nations experience financial constraints.

European surveys indicate that CSP is currently utilized primarily for patients with AV block or following failed left ventricular lead placement. For HF patients with LBBB, traditional BVP remains the preferred strategy among most operators. When CSP is employed, left bundle branch pacing (LBBP) is favored over HBP due to lower pacing thresholds, a shorter procedural learning curve, and a higher success rate for LBBB reversal [13].

However, ensuring persistent LBB capture is critical, as loss of capture can have deleterious effects on HF patients. This necessitates rigorous follow-up. Unfortunately, the current lack of automated capture management algorithms precludes effective remote monitoring (RM), leaving the 12-lead ECG as the only reliable method to confirm capture.

## Resource-specific challenges and barriers

### Financial and procurement hurdles

#### The high cost of specialized pacing systems (leads, sheaths, analyzers) and its impact on patient access and hospital budgeting

CRT is well-established for reducing mortality in HF [14]. However, standard biventricular CRT relies on triple-lead systems, which are significantly more expensive than the single or dual-chamber pacemakers used in CSP. In resource-limited settings, utilizing CSP-based CRT offers a sustainable economic advantage, potentially reducing device costs by nearly 50% compared with traditional methods [15]. Consequently, CSP-based CPP is prioritized for patients who do not require defibrillator therapy. By facilitating LBBP, this approach achieves QRS narrowing and appears to sustain the clinical benefits of CRT based on current observational studies [15]. Although effective CSP-based resynchronization is limited to specific anatomical subsets—such as patients with proximal conduction disease rather than extensive intraventricular delays—it remains a highly efficient option. For example, in patients with atrial fibrillation, effective resynchronization may be achieved using only a cost-effective single-chamber pacemaker.

#### The reliance on donations or repurposed devices and the associated logistical and ethical complexities

While the reuse of medical devices offers a potential solution to the inequity of device availability, concerns regarding sterility and patient safety remain paramount. A validated, feasible protocol has been demonstrated to successfully resterilize these devices [16]. If accepted as a standard of care, this process could underpin global collaboration for device donation, significantly improving healthcare utilization in resource-constrained settings.

### Human resource and expertise gaps

The critical shortage of qualified electrophysiologists, cardiologists, and technical support staff is driven by a profound mismatch between the rising clinical need for cardiac device implantations and the stagnant educational infrastructure available for subspecialty training. In many regions, institutional capacity is so severely restricted that fewer than 10 electrophysiology fellows graduate annually. In Thailand, for example, only approximately 6 fellows graduate each year—an educational throughput that is fundamentally unable to keep pace with the massive volume of patients requiring complex cardiac rhythm interventions.

## Strategic approaches to maximization

### Optimizing implantation protocols for low-resource settings

The “minimalist” approach to cardiac device implantation emphasizes simplified procedural setups and the use of cost-effective alternatives, such as standard pacing systems and basic electrogram recordings, to bypass the need for expensive, specialized equipment. By focusing on strategies that maximize initial success and minimize complications, clinicians can significantly reduce the burden of costly reinterventions. A primary example of this pragmatism is the use of programmer-based pacing techniques as a substitute for standard intracardiac EP maneuvers when verifying left bundle branch (LBB) capture. This method involves systematic analysis of QRS complex morphology across incremental pacing outputs to delineate capture thresholds between the conduction system and the myocardium. Observing the transition from non-selective to selective LBB capture allows for a precise bedside evaluation of lead placement and tissue recruitment, effectively validating the procedure without the immediate necessity of a high-fidelity EP recording system.

### Sustainable training and education models

The implementation of structured pedagogical frameworks—incorporating high-fidelity simulation-based training, longitudinal regional mentorship, and formalized clinical exchange programs—is essential for optimizing the technical proficiency of cardiac electrophysiologists. Given the rapid evolution of EP mapping and ablation technologies, continuous medical education remains an obligatory component of clinical practice. In resource-constrained environments, multinational collaborative initiatives serve as a critical mechanism for resource optimization. Such synergistic, multi-institutional programs facilitate the bidirectional transfer of subspecialty expertise, thereby fostering regional clinical excellence and standardization of quality arrhythmia management.

Regional collaborative networks facilitate the aggregation of multicenter longitudinal data, which is essential for evaluating the real-world efficacy and clinical utility of emerging EP technologies. By synthesizing high-volume datasets across diverse healthcare systems, these partnerships provide a robust framework for assessing regional adoption trends and clinical outcomes. Furthermore, the resulting evidence-based insights enable stakeholders to implement strategic resource optimization, ensuring that sophisticated cardiac interventions are utilized with maximum procedural efficiency and cost-effectiveness.

### Device selection and reprocessing strategies

The establishment of a centralized cardiac device repository facilitates the ethical re-utilization of pulse generators, offering a sustainable mechanism to mitigate medical waste and expand access in resource-limited settings. The existing literature has validated rigorous re-sterilization protocols, demonstrating clinical safety and non-inferiority in infection rates compared with de novo implantations. Given that the prohibitive cost of high-voltage devices remains a primary barrier to therapy, a regional centralized model for device donation is essential for optimizing cost-effectiveness. Potential sources for re-utilization include explanted devices with significant residual battery longevity, such as those removed during pocket infections (where the generator is unaffected), device upgrades, or post-mortem acquisitions.

### Monitoring, follow-up, and future outlook

#### Simplified and accessible patient follow-up

Implementing effective follow-up strategies for CSP currently necessitates a shift away from a total reliance on advanced RM technology, as these platforms lack the specialized diagnostic algorithms required for this niche application. While RM is the standard for conventional devices, its automated sensitivity and threshold-tracking features are primarily designed for myocardial capture and lack the granularity to differentiate between specialized conduction system recruitment and pure myocardial capture. This diagnostic deficit is clinically significant because a subtle Loss of Conduction system capture, which RM might fail to flag, can precipitate worsening HF or electrical dyssynchrony. Consequently, the limited reliability of current RM telemetry in verifying CSP-specific capture thresholds remains a primary barrier to its adoption, particularly in high-dependency patients, such as those with HF and reduced ejection fraction, where maintaining physiological activation is mandatory. In patients undergoing CSP implantation, conventional diagnostic evaluations—including chest radiography and the assessment of cardiac biomarkers such as B-type natriuretic peptide—remain integral components of the standard post-procedural surveillance protocol.

#### The need for local data and registries

**Figure 1.**
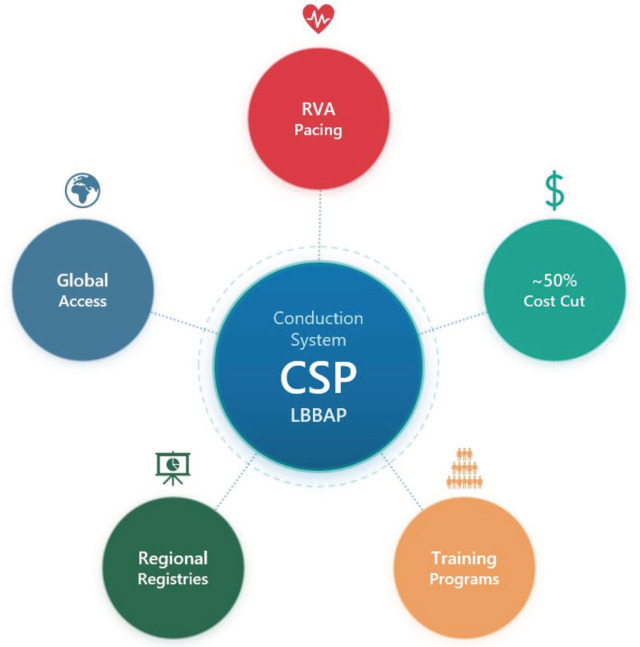
Central illustration: CSP strategy framework for limited resource countries. This figure illustrates a hub-and-spoke model for implementing CSP via LBBAP in limited resource settings. RVA Pacing—Conventional RVA pacing, the current standard in many resource-limited settings, which CSP aims to improve upon by preserving more physiological ventricular activation. Approximately 50% Cost Cut—CSP-based CRT achieves approximately 50% cost reduction compared to conventional triple-lead CRT systems, making advanced HF therapy financially viable in lower-income healthcare systems. Training Programs—Structured education and hands-on training initiatives required to build local operator competency in LBBAP implantation techniques. Regional Registries—Data collection infrastructure to monitor implant outcomes, complication rates, and long-term efficacy across participating centers and geographies. Global Access—The overarching goal of equitable worldwide availability of physiological pacing technology, removing economic and logistical barriers to adoption. CRT, cardiac resynchronization therapy; CSP, conduction system pacing; HF, heart failure; LBBAP, left bundle branch area pacing; RVA, right ventricular apex.

There is a compelling need for the establishment of national and regional registries dedicated to CSP, specifically designed to monitor longitudinal clinical outcomes, procedural complications, and cost-effectiveness within the context of LRCs. The aggregation of granular, local data is instrumental in enhancing the quality of arrhythmia management and serves as a critical foundation for evidence-based financial allocation (**Figure 1**). Furthermore, the development of standardized, clinically relevant metrics is essential for evaluating program efficacy and facilitating continuous quality improvement initiatives across diverse healthcare systems.

### Policy recommendations and collaboration

CSP has emerged as a transformative pacing modality with profound clinical relevance for two primary patient populations: those suffering from AVB and those requiring CRT to manage HF. To fully realize the potential of this physiological pacing approach, there is an urgent call to action for governments, nongovernmental organizations, and medical device manufacturers to actively support the expansion of CSP through subsidized healthcare programs and the development of targeted device technology. By aligning public health policy with industry innovation, stakeholders can bridge the gap in access and ensure that this superior pacing strategy becomes a global standard of care for patients with electrical heart disease.

### Summary and clinical implications

CSP, with a primary focus on LBBAP, represents a transformative shift toward physiological cardiac activation. By maintaining near-physiologic ventricular activation, CSP effectively mitigates the risks of interventricular dyssynchrony and pacing-induced cardiomyopathy associated with traditional RVA pacing. In the context of LRCs like Thailand, CSP offers a sustainable solution to the significant under-penetration of CRT, which is currently hindered by the high cost of triple-lead systems and a shortage of specialized operators trained in coronary venous anatomy. Utilizing CSP-based CRT can potentially reduce device costs by nearly 50%. However, the successful adoption of this modality requires addressing critical infrastructure gaps, including the lack of automated RM for conduction capture and the limited throughput of subspecialty training programs. Ultimately, the integration of minimalist implantation protocols, device reprocessing strategies, and regional longitudinal registries is essential to optimize clinical outcomes and ensure equitable access to advanced arrhythmia management in resource-constrained environments.
